# Whole‐cell studies of substrate and inhibitor specificity of isoprene monooxygenase and related enzymes

**DOI:** 10.1111/1758-2229.13212

**Published:** 2023-11-07

**Authors:** Leanne Sims, Chloe Wright, Andrew T. Crombie, Robin Dawson, Colin Lockwood, Nick E. Le Brun, Laura Lehtovirta‐Morley, J. Colin Murrell

**Affiliations:** ^1^ School of Environmental Sciences University of East Anglia Norwich UK; ^2^ School of Biological Sciences University of East Anglia Norwich UK; ^3^ School of Chemistry University of East Anglia Norwich UK; ^4^ Present address: Quadram Institute Biosciences Norwich Research Park Norwich UK

## Abstract

Co‐oxidation of a range of alkenes, dienes, and aromatic compounds by whole cells of the isoprene‐degrading bacterium *Rhodococcus* sp. AD45 expressing isoprene monooxygenase was investigated, revealing a relatively broad substrate specificity for this soluble diiron centre monooxygenase. A range of 1‐alkynes (C_2_–C_8_) were tested as potential inhibitors. Acetylene, a potent inhibitor of the related enzyme soluble methane monooxygenase, had little inhibitory effect, whereas 1‐octyne was a potent inhibitor of isoprene monooxygenase, indicating that 1‐octyne could potentially be used as a specific inhibitor to differentiate between isoprene consumption by bona fide isoprene degraders and co‐oxidation of isoprene by other oxygenase‐containing bacteria, such as methanotrophs, in environmental samples. The isoprene oxidation kinetics of a variety of monooxygenase‐expressing bacteria were also investigated, revealing that alkene monooxygenase from *Xanthobacter* and soluble methane monooxygenases from *Methylococcus* and *Methylocella*, but not particulate methane monooxygenases from *Methylococcus* or *Methylomicrobium*, could co‐oxidise isoprene at appreciable rates. Interestingly the ammonia monooxygenase from the nitrifier *Nitrosomonas europaea* could also co‐oxidise isoprene at relatively high rates, suggesting that co‐oxidation of isoprene by additional groups of bacteria, under the right conditions, might occur in the environment.

## Graphical abstract

We examined the substrate range of isoprene‐degrading bacteria, and also showed that several microbes which cannot use this major plant‐produced volatile for growth can nevertheless readily degrade it by co‐oxidation. Inhibitor trials identified that octyne and acetylene act as differential inhibitors of isoprene monooxygenase and the monooxygenases of several non‐isoprene degraders. Hence, these inhibitors can be used to distinguish isoprene uptake by *bone fide* isoprene degraders from co‐oxidation by, for example, methane utilizers and nitrifiers.

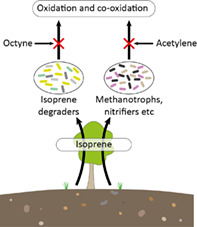

## INTRODUCTION

Isoprene (2‐methyl‐1,3‐butadiene) is an important biogenic volatile organic compound (BVOC) emitted to the atmosphere in quantities similar to that of methane (~500 Tg(C) per year) (Guenther et al., 2012). The vast majority (>90%) is produced by terrestrial plants, possibly in response to heat and oxidative stress (Sharkey et al., 2008) with lesser contributions from bacteria, animals, biomass burning, and industrial processes, reviewed by McGenity et al. (2018). Because of its volatility (boiling point of 34°C) and reactivity caused by the presence of two carbon–carbon double bonds, isoprene has major but multifaceted effects on atmospheric chemistry and therefore on climate (Carlton et al., 2009; Pacifico et al., 2009).

Isoprene‐degrading bacteria can grow aerobically on isoprene as sole carbon and energy source and are a substantial sink for isoprene, removing an estimated 20 Tg of isoprene from the atmosphere per year (Cleveland & Yavitt, 1997; Gray et al., 2015; Pegoraro et al., 2005). Cultivation‐dependent and cultivation‐independent studies have highlighted that isoprene degradation is a widespread trait across particular representatives of many bacterial genera, including *Rhodococcus*, *Mycobacterium*, *Nocardioides*, *Gordonia*, *Sphingopyxis*, and *Variovorax*, and isoprene degraders have been isolated from terrestrial, freshwater, coastal and marine environments including soils and the leaves of trees, reviewed by Dawson et al. (2023). *Rhodococcus* sp. AD45 is a Gram‐positive isoprene‐degrading Actinobacterium isolated from freshwater sediment (van Hylckama Vlieg et al., 1998), and Actinobacteria are often the dominant isoprene degraders in soils or estuarine sediments (El Khawand et al., 2016; Johnston et al., 2017). Proteobacterial isoprene degraders, including *Variovorax*, *Sphingopyxis*, and *Pseudomonas*, have been identified in other environments (Dawson et al., 2020; Gibson et al., 2021; Larke‐Mejía et al., 2019; Srivastva et al., 2015). All characterised isoprene‐degrading bacteria (with the exception of an *Alcaligenes* strain described by Uttarotai et al. (2022)) contain 11 core *iso* genes clustered together. An isoprene monooxygenase (IsoMO) is encoded by *isoABCDEF* while the gene products of *isoGHIJ* and *aldH* are required for subsequent metabolic steps (Dawson et al., 2022; van Hylckama Vlieg et al., 2000). In *Rhodococcus* sp. AD45 and *Variovorax* sp. WS11, *iso* genes for isoprene metabolism are located on megaplasmids (Crombie et al., 2015; Dawson et al., 2020) suggesting that the ability to grow on isoprene may be acquired via horizontal gene transfer.

In all strains where the isoprene metabolic pathway has been identified, isoprene is initially oxidized to epoxyisoprene, catalysed by IsoMO (Sims et al., 2022; van Hylckama Vlieg et al., 2000), which is a member of the soluble diiron monooxygenase (SDIMO) family that includes soluble methane monooxygenase (sMMO), alkene and aromatic monooxygenases and phenol hydroxylases (reviewed by Leahy et al. (2003)). Alignments of the alpha‐oxygenase component of IsoMO (IsoA), which contains the conserved diiron active site, with equivalent alpha‐oxygenase components from other SDIMOs indicate that among these IsoMO is most closely related to the four‐component alkene/aromatic monooxygenases, for example, alkene MO from *Xanthobacter autotrophicus* Py2 or toluene monooxygenase from *Pseudomonas stutzeri* OX1 (Sazinsky et al., 2004; Small & Ensign, 1997; van Hylckama Vlieg et al., 2000). IsoMO is a four‐component SDIMO comprised of an α_2_β_2_γ_2_ oxygenase (IsoABE), a Rieske‐type ferredoxin (IsoC), a coupling/effector protein (IsoD), and an NAD^+^ reductase (IsoF). In vitro experiments with IsoMO subunits from *Rhodococcus* sp. AD45 showed that all components are necessary for activity (Sims et al., 2022). Following oxidation of isoprene to epoxyisoprene, a glutathione‐*S*‐transferase (IsoI) conjugates epoxyisoprene with glutathione (GSH) to produce 1‐hydroxy‐2‐glutathionyl‐2‐methyl‐3‐butene (HGMB), detoxifying the cell from the reactive epoxide. From HGMB, 2‐glutathionyl‐2‐methyl‐3‐butenoate (GMBA) is produced via a dehydrogenase (IsoH), before incorporation of carbon into central metabolism via the beta‐oxidation pathway (Dawson et al., 2022; Rix et al., 2023).

Co‐oxidation is the transformation of a non‐growth substrate which, due to a lack of substrate specificity, can nevertheless serve as substrate for an enzymatic process during normal metabolism (Dalton et al., 1982). For example, bacterial SDIMOs typically co‐oxidise a wide array of alternative substrates. Dawson et al. (2020) investigated the substrate range of IsoMO in *Variovorax* sp. WS11 and showed that this isoprene degrader could co‐oxidise a broad range of alkenes, with preference for those with alkyl side chains. sMMO from methanotrophs transforms an extensive range of over 100 non‐growth substrates including C_2_–C_9_ alkanes and C_2_–C_5_ alkenes, halogenated hydrocarbons, and aromatics such as styrene and toluene (Colby et al., 1977; Jiang et al., 2010; Smith & Dalton, 2004). Alkene monooxygenase from *X. autotrophicus* Py2 can catalyse the epoxidation of a range of alkenes, including trichloroethylene, and aromatics, but not alkanes (van Ginkel et al., 1987; Zhou et al., 1999). Some SDIMO‐containing strains, including methanotrophs, Actinobacteria and Gram‐negative toluene degraders, oxidise isoprene or 1,3‐butadiene, which is structurally similar to isoprene (McClay et al., 2000; Patel et al., 1982; van Ginkel & de Bont, 1986), suggesting that some non‐isoprene‐degraders may contribute to isoprene consumption in the environment.

Copper‐dependent membrane monooxygenases (CuMMO), such as particulate methane monooxygenase (pMMO) and ammonia monooxygenase (AMO), are also ‘promiscuous’ in terms of substrate range. pMMO oxidises fewer alternative substrates compared to sMMO, including C_2_–C_5_ alkanes, C_2_–C_4_ alkenes and some, halogenated hydrocarbons, but not aromatic compounds (Burrows et al., 1984; Lontoh et al., 2000). AMO from *Nitrosomonas europaea* co‐oxidises over 40 substrates including C_2_–C_8_ alkanes, C_2_–C_5_ alkenes, halogenated hydrocarbons, and aromatics (Hyman et al., 1988; Keener & Arp, 1993; Keener & Arp, 1994), making it more similar to sMMO in terms of its co‐oxidative abilities.

While co‐oxidative processes are of biotechnological interest for their ability to generate high‐value compounds including desirable enantiomers (Smith & Dalton, 2004), and although bacteria growing on isoprene probably constitute the major biological sink for isoprene, due to the ubiquity of sMMO, pMMO, and AMO‐containing microorganisms in the environment, their potential contribution to the microbial sink for isoprene via co‐oxidation needs to be addressed. The use of specific inhibitors can aid these studies. For example, sMMO, pMMO, and AMO are inhibited by acetylene (Hynes & Knowles, 1982; Prior & Dalton, 1985), alkene monooxygenases from *Xanthobacter autotrophicus* Py2 and *Rhodococcus rhodochrous* B276 are inhibited by propyne (Ensign et al., 1992; Fosdike et al., 2005), and toluene monooxygenases are inhibited by aromatic alkynes with terminal acetylene groups (Keener et al., 2001). Previous data from our lab showed that, compared to acetylene, IsoMO from *Variovorax* sp. WS11 was inhibited more by longer chain 1‐alkynes such as 1‐octyne (Dawson et al., 2020), which may provide a starting point for identification of a specific inhibitor of IsoMO.

This study aimed to characterize the whole‐cell substrate specificity of IsoMO from *Rhodococcus* sp. AD45 and the effects of potential inhibitors such as C_2_–C_8_ linear 1‐alkynes. We wished to compare these to the inhibition profiles of *Variovorax* sp. WS11 and a methanotroph expressing sMMO, *Methylococcus capsulatus* strain Bath. Furthermore, we determined the rates of isoprene oxidation by methanotrophs (*Methylocella*, *Methylococcus*, *Methylomicrobium*) and ammonia oxidisers (*Nitrosomonas*), and the kinetic parameters of isoprene oxidation by *Rhodococcus* sp. AD45, *Variovorax* sp. WS11 and *X. autotrophicus* Py2 in comparison with the kinetics of propylene uptake in order to expand our knowledge of the substrate range of isoprene monooxygenase, its inhibitors, and the potential for co‐oxidation of isoprene by non‐isoprene‐degraders.

## EXPERIMENTAL PROCEDURES

### Strains and growth media

*Rhodococcus* sp. AD45 and *Variovorax* sp. WS11 were grown in their respective media in batch mode in a 4‐L working volume fermentor as previously described (Crombie et al., 2015; Dawson et al., 2020). *Xanthobacter autotrophicus* Py2 and *Rhodococcus* sp. AD45 (for epoxyisoprene product formation) were grown in wash culture in minimal medium as described (Crombie et al., 2015) and supplied with isoprene (approx. 1% v/v) or propylene (10% v/v) in the headspace. *Methylococcus capsulatus* (Bath) was grown in nitrate mineral salts medium (Whittenbury et al., 1970) as previously described (Dawson et al., 2020) and *Methylomicrobium alcaliphilum* in modified P medium (Akberdin et al., 2018) in 50 mL volumes in 250 mL Quickfit conical flasks, shaking (180 rpm) at 30°C and supplied with methane (20% v/v). For expression of sMMO, *Methylococcus capsulatus* was grown in copper‐free medium, and the naphthalene assay was used to verify sMMO expression (Brusseau et al., 1990). *Nitrosomonas europaea* ATCC19718 was grown in modified Skinner and Walker medium as described (Wright et al., 2020). Cells were harvested by centrifugation (except for *Nitrosomonas europaea* which was harvested by filtration as described; Wright et al., 2020), washed and resuspended in the buffer specified below and either used fresh or drop‐frozen in liquid nitrogen and stored at −80°C.

### Rhodococcus sp. AD45 substrate range

Substrate‐induced oxygen uptake was quantified using a Clark‐type oxygen electrode, as previously described (Dawson et al., 2020). Potential substrates were prepared as aqueous solutions in sealed flasks. In the case of gaseous substrates, aqueous concentrations were calculated from the headspace concentration using values of the Henry's law constants taken from Sander (2015). Cell suspension (1.5 mg dry weight of cells; dw)) in a volume of 3 mL air‐saturated phosphate buffer (50 mM, pH 7.0) was added to the reaction cell, maintained at 30°C. After recording the endogenous rate of oxygen uptake, substrates (5–150 μL) were injected into the chamber to a final concentration of 100 μM. The substrate‐induced rate was calculated by subtracting the endogenous rate from the rate in the presence of substrate.

### Inhibition of IsoMO with terminal alkynes

The inhibition of IsoMO by 1‐alkynes was quantified as in Dawson et al. (2020). Briefly, approximately 0.25 mg (dw) of *Rhodococcus* sp. AD45 cells were resuspended in 1 mL phosphate buffer (50 mM, pH 7.0) in a 25 mL flask, sealed with a rubber stopper, and incubated at 30°C in a shaking water bath. Isoprene (300 ppmv) was added by injection through the septum as previously described (Crombie et al., 2015) and left to equilibrate for 3 min. Isoprene was quantified from headspace samples every 5 min. After 15 min, inhibitors were added to a final concentration of 50 μM. Gaseous 1‐alkyne inhibitors (C_2_–C_4_) were added directly to assay vials using a gas‐tight syringe and the resultant aqueous concentration calculated using the Henry's law coefficient as described above. C_5_–C_8_ (liquid) alkynes were dissolved in DMSO and added in a volume of 10 μL, having previously established that this volume of DMSO alone had no effect on isoprene oxidation by IsoMO. After allowing 5 min for the reaction to stabilise, a further five headspace samples were taken at 5 min intervals. Rates of isoprene uptake in the presence or absence of alkyne inhibitors were quantified by injection of 50 μL of headspace gas into an Agilent 7820A gas chromatograph fitted with a Porapak Q column (Supelco) coupled to a flame ionisation detector. The injector, oven, and detector temperatures were 150, 125, and 200°C, respectively. Headspace isoprene concentration was quantified using standards prepared from a known quantity of isoprene in air. The percentage inhibition was calculated by comparing the change in isoprene uptake before and after the addition of inhibitor. Control experiments with no inhibitor added or with addition of DMSO alone were used to account for any reduction in rate due to the decreasing isoprene concentration.

### Kinetics of isoprene and propylene uptake

Frozen cells of *Rhodococcus* sp. AD45 or *X. autotrophicus* Py2 (0.25–3.5 mg dw) were resuspended in 1–2 mL phosphate buffer (50 mM, pH 7.0) in 25 mL vials, sealed with rubber stoppers and incubated at 30°C in a shaking water bath. For isoprene uptake by *X. autotrophicus* Py2, pyruvate (10 mM) was added as a reductant. Isoprene or propylene (140–40,000 ppmv) was added to the headspace and uptake measured by injection of headspace gas (10–100 μL) into a fast isoprene sensor (Hills Scientific, Boulder, CO, USA) every 3–15 min, against standards prepared in the range 12–40,000 ppmv. Corresponding liquid phase concentrations were calculated using the Henry's law constants as described above. Data were analysed using QtiPlot (v 5.6.1) and gas uptake rates plotted against substrate concentration. Fermentor‐grown cells of *Variovorax* sp. WS11 (0.25 mg dw) were transferred to 30 mL vials, sealed and isoprene vapour was added to the headspace. The rate of isoprene uptake was measured for 15 min using a gas chromatograph as described above. The apparent *K*_*m*_ and *V*_max_ for isoprene or propylene uptake, together with associated standard error and 95% confidence interval, were calculated by non‐linear regression using Hyper32 (v. 1.0) software (University of Liverpool, UK).

### Detection of epoxyisoprene by GC–MS

Cells were resuspended in buffer (5 mL), at the growth pH for each strain, to a final OD_540_ between 0.25 and 6.0 in 25 mL glass vials, sealed with rubber stoppers. To inhibit monooxygenase activity, the cells were preincubated with alkynes for 30 min at their respective growth temperatures. For sMMO and CuMMOs, acetylene was added to a concentration of 0.04 mM and, for the alkene MO and IsoMO, 1‐propyne (9.2 mM) and 1‐octyne (0.05 mM), respectively. The cells were then washed twice and resuspended in alkyne‐free buffer with additional reductant if required (20 mM formate for methanotrophs or 0.6 mM hydrazine for *N. europaea*). For *R*. sp. AD45, epoxyhexane (1 mM) was added to inhibit subsequent metabolism of epoxyisoprene (van Hylckama Vlieg et al., 1998). An initial sample (0.4 mL) was taken, isoprene vapour was added to a liquid concentration of approximately 130 μM and vials were incubated with shaking. Cell suspension (0.4 mL) was removed with a syringe through the septum every 15 min and transferred to a 1.5 mL tube. Epoxyisoprene was extracted into diethyl ether alongside standards prepared from known concentrations (0.002–4.0 mM) of commercial epoxyisoprene (Merck, UK) in diethyl ether and quantified by gas chromatography (GC)–MS as previously described (Rix et al., 2023).

## RESULTS AND DISCUSSION

### The substrate specificity of Rhodococcus sp. AD45

**FIGURE 1:**
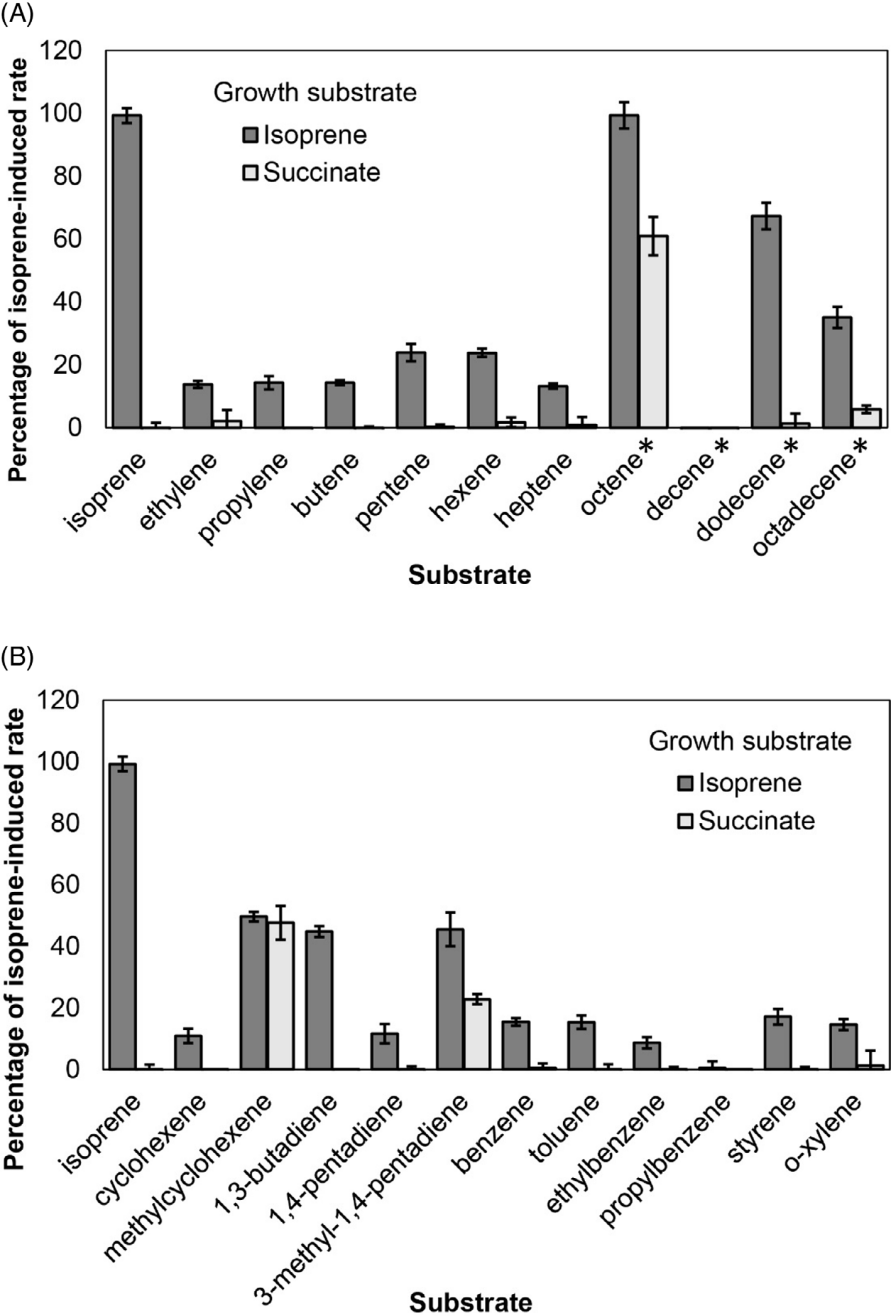
Relative rates of substrate‐induced oxygen uptake by whole‐cell suspensions of isoprene‐grown (dark grey bars) or succinate‐grown (light grey bars) *Rhodococcus* sp. AD45. Rates are presented as a percentage of the rate of isoprene‐induced oxygen uptake by isoprene‐grown cells. Oxygen uptake rate induced by (A) terminal alkenes and (B) dienes, cyclic alkenes, and aromatics. Error bars show standard error of the mean (*n* = 3). *, low‐solubility substrate.

*Rhodococcus* sp. AD45 was grown in batch culture on either isoprene or succinate, conditions under which IsoMO is either expressed or repressed, respectively (Crombie et al., 2015). Rates of alkene‐induced oxygen uptake were quantified using a Clark‐type oxygen electrode. Substrates were added to 100 μM, or to the limit of solubility for less soluble longer‐chain alkenes (as indicated in Figure 1). With the exception of the compounds mentioned below, oxygen uptake was not stimulated by alkenes in succinate‐grown *Rhodococcus* sp. AD45 cells (Figure 1). However, IsoMO‐expressing cells oxidised a wide range of alkenes without a distinct relationship between the length or structure of the alkene and the resultant rate. The specific rate of oxygen uptake in response to isoprene was 23.1 ± 0.4 nmol min^−1^ mg dw^−1^, whereas 1,3‐butadiene, which relative to isoprene lacks only a methyl group, induced oxygen uptake at half the rate of isoprene (10.4 ± 0.3 nmol min^−1^ mg dw^−1^) (Figure 1B). 1‐octene induced a near identical rate to that of isoprene, by cells expressing IsoMO (23.1 ± 0.8 nmol min^−1^ mg dw^−1^), but there was also a considerable rate of 1‐octene‐induced oxygen uptake by succinate‐grown cells (14.2 ± 1.6 nmol min^−1^ mg dw^−1^). Similarly, methylcyclohexene and 3‐methyl‐1,4‐pentadiene also induced oxygen uptake by succinate‐grown cells, suggesting that these compounds may also be substrates for a different, constitutively expressed oxygenase. Apart from IsoMO, the genome of the metabolically versatile *Rhodococcus* sp. AD45 encodes numerous oxygenases and other enzymes involved in the metabolism of aliphatic and aromatic hydrocarbons (including several cytochrome P450), some of which may be expressed during growth on succinate (Crombie et al., 2015). The data (Figure 1A) show that these are inactive with isoprene, but some could be active with alternative compounds tested here. Also, note that *bona‐fide* isoprene degraders will consume more oxygen per mole isoprene metabolized than organisms only able to complete the initial oxygenation since the latter will be unable to further oxidize the resultant epoxide. Thus, the stoichiometry and relative rates of oxygen and isoprene uptake might differ between these two groups, possibly resulting in a slight underestimation of the relative rate of isoprene consumption by a co‐oxidiser.

The substrate profile of isoprene‐grown *Rhodococcus* sp. AD45 was broadly similar to that of *Variovorax* sp. WS11 (Dawson et al., 2020), albeit with some differences. Unlike *Rhodococcus* sp. AD45, *Variovorax* sp. WS11 did not respond to cyclohexene or benzene as substrates but could oxidise alkylated versions (Dawson et al., 2020). Furthermore, 1‐octene did not induce oxygen uptake by *Variovorax* sp. WS11 cells grown on succinate. Alkene monooxygenase (alkene MO) from *X. autotrophicus* Py2 (of which the alpha subunit shares 70% amino acid identity with IsoA from *R*. sp. AD45) oxidises many of the same substrates as *Rhodococcus* sp. AD45 and *Variovorax* sp. WS11, including ethylene, propylene, 1‐butene, *cis* and *trans‐*2‐butene, 1,3‐butadiene, 1‐pentene, and 1‐hexene (van Ginkel et al., 1987). The related SDIMOs toluene 2‐monooxygenase and toluene 4‐monooxygenase from *Burkholderia cepacia* G4 and *Pseudomonas mendocina* KR1, respectively, were capable of oxidizing 1,3‐butadiene, 2‐butene, 1‐pentene, 2‐pentene, 2‐chloropropene and 2,3‐dichloropropene (McClay et al., 2000). These data suggest that enzymes from this group of SDIMOs [Group I according to the classification of Holmes and Coleman (2008)] share a broad substrate specificity for alkenes.

### Inhibition of oxygenase activity by alkynes

Triple‐bonded compounds used in metalloenzyme inhibition studies include acetylene and longer‐chain alkynes. For example, acetylene inhibits many metalloenzyme‐catalysed microbial processes including N_2_ fixation, denitrification, nitrification, methanotrophy, methanogenesis, nitrate assimilation, and H_2_ metabolism [reviewed in Hyman and Daniel (1988)] and acts as a suicide substrate for sMMO, pMMO, and AMO (Hyman & Wood, 1985; Hynes & Knowles, 1982; Prior & Dalton, 1985). However, for sMMO, longer chain‐length alkynes including propyne and 1‐butyne are not as inhibitory as acetylene (Dalton & Whittenbury, 1976; Stirling & Dalton, 1977). The sensitivity of *Rhodococcus* sp. AD45 to C_2_–C_8_ linear 1‐alkynes was tested and compared to previously published data for *Variovorax* sp. WS11 and sMMO‐expressing *M. capsulatus* (Dawson et al., 2020). Isoprene was added to a liquid‐phase concentration of 5 μM and uptake from the headspace was measured by GC. All alkynes were added to a final concentration of 50 μM, with C_5_–C_8_ alkynes dissolved in DMSO, which alone at the concentration used had no inhibitory effect on oxygenase activity in the strains reported here.

**FIGURE 2:**
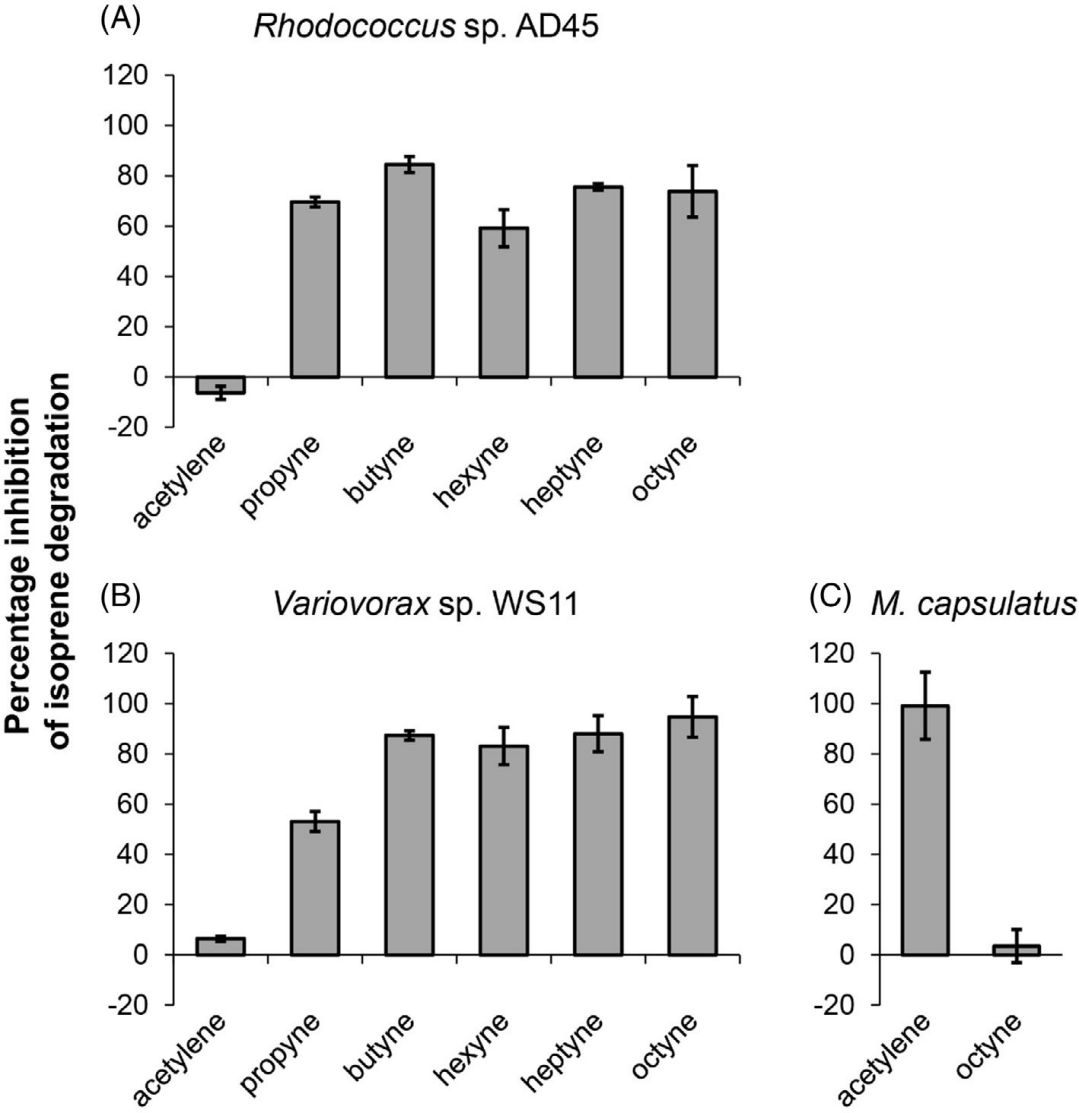
Inhibition of isoprene oxidation by whole cells of (A) isoprene‐grown *Rhodococcus* sp. AD45, (B) isoprene‐grown *Variovorax* sp. WS11, or (C) sMMO‐expressing *M. capsulatus*, in response to 50 μM C_2_–C_8_ linear 1‐alkynes. *Variovorax* sp. WS11 and *M. capsulatus* data from Dawson et al. (2020). Error bars show standard error of the mean (*n* = 3).

Acetylene had no inhibitory effect on isoprene degradation by *Rhodococcus* sp. AD45, whereas C_4_–C_8_ 1‐alkynes had a significant inhibitory effect and 1‐octyne inhibited isoprene uptake by 74% (Figure 2A). This is similar to the data reported for *Variovorax* sp. WS11, which was inhibited by acetylene and 1‐octyne by 6.5% and 95%, respectively (Dawson et al., 2020), Figure 2B. In contrast, isoprene oxidation by the methanotroph *Methylococcus capsulatus*, expressing sMMO, was inhibited by 99% and 5.7% by acetylene and 1‐octyne, respectively (Dawson et al., 2020), Figure 2C. The increasingly potent inhibitory effect of longer chain alkynes, as compared to acetylene, was also found for growth of alkene MO‐containing *Rhodococcus rhodochrous* B276 (Fosdike et al., 2005; Gallagher et al., 1997), and propyne was found to be an effective inhibitor of alkene MO from *X. autotrophicus* Py2, whereas acetylene had no effect (Ensign et al., 1992). For toluene 4‐monooxygenase, which is closely related to IsoMO (Crombie et al., 2015), acetylene was ineffective compared with aliphatic or aromatic alkynes (Yeager et al., 1999). It would be interesting to test the effects of aromatic alkynes on IsoMO activity, as differences in cellular architecture and physiology may cause variations in the effectiveness of alkyne inhibitors.

### The production of epoxyisoprene from isoprene by monooxygenases

We assessed whether other monooxygenases, including the membrane‐bound copper CuMMO, could oxidise isoprene during whole‐cell activity assays by measuring epoxyisoprene formation from isoprene. Methane oxidisers and the nitrifier *N. europaea*, expressing AMO, were supplemented with formate or hydrazine, respectively, as external reductant. For the isoprene degrader *Rhodococcus* sp. AD45, epoxyhexane, an irreversible inhibitor of the glutathione *S*‐transferase IsoI, was added to cell suspensions to prevent the subsequent metabolism of the epoxyisoprene generated (van Hylckama Vlieg et al., 1998). The rate of epoxyisoprene production was quantified using GC–MS (Table 1).

**TABLE 1:** Initial rates of epoxyisoprene production from isoprene.

Strain (oxygenase expressed)	Rate of epoxyisoprene formation (nmol min^−1^ mg dw^−1^)	Epoxyisoprene formation in the presence of the oxygenase inhibitor (in parentheses)
*Rhodococcus* sp. AD45 (IsoMO)	1.44 (0.06)	n.d. (1‐octyne)
*X. autotrophicus* Py2 (alkene MO)	1.82 (0.04)	n.d. (1‐propyne)
*Methylocella silvestris* (sMMO)	1.37 (0.03)	n.d. (acetylene)
*Methylococcus capsulatus* (sMMO)	1.29 (0.03)	n.d. (acetylene)
*Methylomicrobium alcaliphilum* (pMMO)	0.05 (0.01)	n.d. (acetylene)
*Methylococcus capsulatus* (pMMO)	0.08 (0.01)	n.d. (acetylene)
*Nitrosomonas europaea* (AMO)	4.80 (0.49)	n.d. (acetylene)

*Note*: The rates shown are the mean of three replicates ± standard error of the mean.

Abbreviation: n.d., not detected.

The rates of epoxyisoprene production from isoprene by cells expressing SDIMOs were similar (1.38–1.88 nmol min^−1^ mg dw^−1^) but contrasted with methanotrophs expressing pMMO (Table 1), which were 20‐fold less for both *M. alcaliphilum* and *M. capsulatus*. Curiously, AMO from *N. europaea* (a CuMMO similar to pMMO of methanotrophs) had the fastest rate of all the oxygenases tested, approximately three times faster than IsoMO from *Rhodococcus* sp. AD45. However, since epoxides are highly reactive and toxic to the cell, it is likely that the accumulation of epoxyisoprene would prevent high rates being sustained for longer periods in non‐isoprene degraders. Epoxyisoprene production by the different bacterial strains was efficiently inhibited by the alkynes specific to the oxygenase expressed. For sMMO and CuMMOs acetylene was used, for *Rhodococcus* sp. AD45 and *X. autotrophicus* Py2 1‐octyne and 1‐propyne, respectively (Table 1).

### Isoprene and propylene uptake kinetics by whole cells

To examine the specificity of IsoMO and alkene MO for isoprene and propylene, uptake kinetic parameters were determined for whole cells of the isoprene degraders *Rhodococcus* sp. AD45 and *Variovorax* sp. WS11, and the propylene degrader *X*. *autotrophicus* Py2, grown on isoprene or propylene, respectively, by measuring the rate of depletion of headspace gas. Isoprene and propylene uptake by *Rhodococcus* sp. AD45, *Variovorax* sp. WS11 and *X. autotrophicus* Py2 followed Michaelis–Menten type saturation kinetics. The *K*_*m*(app)_ and *V*_max(app)_ for isoprene determined here for *Rhodococcus* sp. AD45 were 4.0 μM and 47.2 nmol min^−1^ mg dw^−1^, respectively (Table 2), in reasonably good agreement with the results of van Hylckama Vlieg et al. (1998). Cells of *Variovorax* sp. WS11 had a four‐fold lower affinity (10.5 μM) but a similar maximum rate, comparable with values reported for the isoprene degrader *Sphingopyxis* sp. OPL5 (Larke‐Mejía et al. (2020), Table 2). Interestingly, *Rhodococcus* sp. AD45 had a similar affinity and maximum oxidation rate for its non‐growth substrate, propylene, as for isoprene (*K*_*m*(app)_ (propylene) and *V*_max(app)_ (propylene) of 2.0 μM and 36.6 nmol min^−1^ mg dw^−1^ respectively) (Table 2). *X. autotrophicus* Py2 had a similar affinity for propylene (*K*_*m*(app)_ 5.0 μM), its growth substrate, as *Rhodococcus* sp. AD45 and only a moderately higher maximum rate, (*V*_max(app)_ 97.2 nmol min^−1^ mg dw^−1^) (Table 2). However, its affinity for isoprene was more than 10‐fold lower (*K*_*m*(app)_ 55.1 μM), consistent with the fact that isoprene is not its natural substrate.

**TABLE 2:** Isoprene and propylene uptake kinetics of isoprene degraders, in comparison with *X. autotrophicus* Py2.

Strain (oxygenase)	*K*_m(app)_ (μM)	*V*_max(app)_ (nmol min^−1^ mg dw^−1^)	Reference
Isoprene			
*Rhodococcus* sp. AD45 (IsoMO)	**4.0 ± 1.3 (0.6–7.4)**	**47.2 ± 5.2 (33.8–60.6)**	This study
	0.8	76	van Hylckama Vlieg et al. (1998)
*X. autotrophicus* Py2 (alkene MO)	**55.1 ± 24.4 (0.6–109.6)**	**22.1 ± 2.7 (16.1–28.0)**	This study
*Sphingopyxis* sp. OPL5 (IsoMO)^a^	2.5	10	Larke‐Mejia et al. (2020)
*Variovorax* sp. WS11 (IsoMO)	**10.5 ± 3.9 (1.3–19.7)**	**35.4 ± 5.7 (21.9–48.9)**	This study
Propylene			
*Rhodococcus* sp. AD45 (IsoMO)	**2.0 ± 0.8 (0.0–4.0)**	**36.6 ± 6.2 (20.5–52.6)**	T**h**is study
*X. autotrophicus* Py2 (alkene MO)	**5.0 ± 1.2 (1.8–8.3)**	**97.2 ± 10.4 (68.2–126.2)**	This study
	0.6^b^–1.3	70^c^–75	van Ginkel and de Bont (1986); Reij et al. (1995)

*Note*: The data from this study, (shown in bold font ± standard error (95% confidence interval), are compared with previous studies.

^a^ Based on substrate‐induced oxygen uptake.

^b^ Converted from gas phase concentration.

^c^ Rate given as per mg protein.

## CONCLUSIONS

In summary, using whole‐cell studies, we show that isoprene monooxygenase from the Gram‐positive *Rhodococcus* sp. AD45 is a catalytically versatile member of the SDIMO enzyme family, which co‐oxidises a wide range of alkenes, dienes and even aromatic compounds, consistent with findings in the Gram‐negative *Variovorax*. This demonstrates that isoprene monooxygenases from distinctly different groups of microorganisms, with relatively modest levels of sequence identity, are functionally similar in terms of their inhibition and co‐oxidation profiles. IsoMO generates isoprene epoxide with a high enantiomeric excess (Dawson et al., 2023) and the biotechnological potential of this enzyme should be explored further for the production of bulk and high value compounds such as chiral epoxides. We demonstrate that particulate methane monooxygenase cannot co‐oxidise isoprene but that soluble methane monooxygenase, and surprisingly, ammonia monooxygenase from *Nitrosomonas*, can co‐oxidise isoprene at significant rates. This has implications for alternative routes of isoprene removal/degradation by the multiple different monooxygenases represented in microbial communities. For example, peat uplands are significant sources of both methane and isoprene and methanotrophs are abundant in these environments (Chen et al., 2008; Faubert et al., 2011). Currently, however, the extent to which methanotrophs contribute to isoprene degradation (and vice versa) is unknown. Similarly, estuarine and coastal regions are considerable sources of isoprene, while harbouring diverse nitrifier communities (Bernhard & Bollmann, 2010; Exton et al., 2012), also with an unknown impact on isoprene biodegradation. This co‐metabolic activity may also supply carbon to the wider community via hydrolysis of the relatively unstable epoxide (Gervasi & Longo, 1990). While the data presented here suggest that these effects may not be negligible, we also provide a toolkit for its quantification. We show that 1‐octyne is a potent inhibitor of isoprene monooxygenase and that acetylene, which inhibits oxygenases such as methane and ammonia monooxygenase, does not significantly inhibit isoprene monooxygenase. Thus, parallel incubations of environmental samples, either un‐amended or following inhibition with either octyne or acetylene, provide a method to evaluate the contribution of these co‐oxidisers to isoprene degradation. This has important implications for future environmental studies which seek to distinguish the relative roles of isoprene degradation by bona fide isoprene‐utilising bacteria from co‐oxidation by other groups such as methanotrophs and nitrifiers.

## AUTHOR CONTRIBUTIONS

**Leanne Sims:** Conceptualization (equal); investigation (lead); methodology (equal); writing – original draft (equal); writing – review and editing (equal). **Chloe Wright:** Conceptualization (equal); investigation (lead); methodology (equal); writing – original draft (equal); writing – review and editing (equal). **Andrew Crombie:** Conceptualization (supporting); investigation (supporting); methodology (supporting); writing – original draft (equal); writing – review and editing (equal). **Robin Dawson:** Conceptualization (supporting); investigation (equal); methodology (supporting); writing – original draft (supporting); writing – review and editing (equal). **Colin Lockwood:** Conceptualization (supporting); investigation (supporting); methodology (supporting); writing – review and editing (supporting). **Nick Le Brun:** Conceptualization (supporting); funding acquisition (supporting); methodology (supporting); writing – review and editing (equal). **Laura Lehtovirta‐Morley:** Funding acquisition (supporting); supervision (supporting); writing – review and editing (equal). **J. Colin Murrell:** Conceptualization (lead); funding acquisition (lead); methodology (supporting); project administration (lead); resources (equal); supervision (equal); writing – original draft (equal); writing – review and editing (equal).

## CONFLICT OF INTEREST STATEMENT

The authors declare no conflict of interest.

## Data Availability

All data generated and analysed during this study are included in this published article and its supplementary information files.
